# Explaining Spatial Heterogeneity in Population Dynamics and Genetics from Spatial Variation in Resources for a Large Herbivore

**DOI:** 10.1371/journal.pone.0047858

**Published:** 2012-10-31

**Authors:** Adrienne L. Contasti, Emily J. Tissier, Jill F. Johnstone, Philip D. McLoughlin

**Affiliations:** Department of Biology, University of Saskatchewan, Saskatoon, Saskatchewan, Canada; Ben-Gurion University of the Negev, Israel

## Abstract

Fine-scale spatial variation in genetic relatedness and inbreeding occur across continuous distributions of several populations of vertebrates; however, the basis of observed variation is often left untested. Here we test the hypothesis that prior observations of spatial patterns in genetics for an island population of feral horses (Sable Island, Canada) were the result of spatial variation in population dynamics, itself based in spatial heterogeneity in underlying habitat quality. In order to assess how genetic and population structuring related to habitat, we used hierarchical cluster analysis of water sources and an indicator analysis of the availability of important forage species to identify a longitudinal gradient in habitat quality along the length of Sable Island. We quantify a west-east gradient in access to fresh water and availability of two important food species to horses: sandwort, *Honckenya peploides*, and beach pea, *Lathyrus japonicas*. Accordingly, the population clusters into three groups that occupy different island segments (west, central, and east) that vary markedly in their local dynamics. Density, body condition, and survival and reproduction of adult females were highest in the west, followed by central and east areas. These results mirror a previous analysis of genetics, which showed that inbreeding levels are highest in the west (with outbreeding in the east), and that there are significant differences in fixation indices among groups of horses along the length of Sable Island. Our results suggest that inbreeding depression is not an important limiting factor to the horse population. We conclude that where habitat gradients exist, we can anticipate fine-scale heterogeneity in population dynamics and hence genetics.

## Introduction

It is now clear that over continuous distributions, spatial variability in individual behaviour can lead to small-scale spatial heterogeneity in movements [1]. Where this occurs, fine-scale genetic substructure may arise, which has importance for understanding kin relationships and genetic diversity of populations [2], [3], [4], [5], [6]. For example, localization of movements and limited dispersal may result in close spatial associations between relatives and increase instances of inbreeding [7]. Similarly, spatial variation in the age- and sex-structure of groups of competing individuals in polygynous species [8], [9] may contribute to spatial variation in monopolization of mating opportunities, with resulting effects on genetic substructure [10].

The processes that cause dispersal to be limited or lead to variation in mating structures have not been well addressed in the context of fine-scale population substructure, though social structure, limited dispersal, and spatial heterogeneity in resources are common in wild vertebrate populations [6]. Clustering of important resources or attractants including food, water, and predator refugia; social friction (e.g., escalated conflicts between strangers [11]); and physical barriers to movement (e.g., habitat fragmentation) are possible explanations for irregularities in movements and thus demography across a population's otherwise continuous range. Demonstrating why a population might vary in demography, and thus genetic relatedness and inbreeding, may further the development of new theory (e.g., evolution of specialists and generalists in heterogeneous habitats [12]) but also bring to attention potential problems for conservation and management (e.g., confounding effects on population and quantitative genetics [2], [6]).

Recently, Lucas et al. [13] demonstrated a linear (longitudinal) gradient in fine-scale genetic substructure for the feral horses (*Equus ferus caballus*) of Sable Island, Nova Scotia, Canada. Across four equal-sized divisions of the long (49 km) and narrow (<1.5 km) island, heterozygosity ranged from a low of 0.589 to a high of 0.694 across a gradient from west to east. Pairwise fixation indices (*F*_ST_ [14]) were significantly different for most subdivision pairs in the analysis, ranging as high as 0.067 from the west side of the island to the east. Western areas showed highest levels of inbreeding (*F*_IS_ = 0.113 [15]), with outbreeding indicated in the east (*F*_IS_ = −0.008).

Here we test the hypothesis that the observed spatial variation in genetic substructure of the Sable Island horses was due to a west-east gradient in spatial variation in habitat quality with resultant effects on demography. We first determined if a longitudinal gradient in resources thought to be important for horses, including fresh water and high-quality forage, existed along the length of Sable Island. We then determined if our observations concerning habitat quality could explain spatial variation in demographic and biological parameters (population density, body condition, survival, and reproduction) and movement patterns (population clustering). We expected that where habitat quality was high, inbreeding should also be high, as females and their offspring may be attracted to high quality sources of food and/or water (and vice-versa, in poor-quality areas). We found that both gradients in habitat quality and demography can explain previous findings in terms of population genetics. We conclude that where habitat gradients exist, researchers should anticipate fine-scale heterogeneity in population dynamics and thus genetics.

## Methods

### Ethics statement

We thank Canada Coast Guard for granting us access to Sable Island for the purpose of our research. Sampling was carried out under University of Saskatchewan Animal Care Protocol 20090032 and under guidance of the Canadian Council on Animal Care.

### Study area

**Figure 1:**
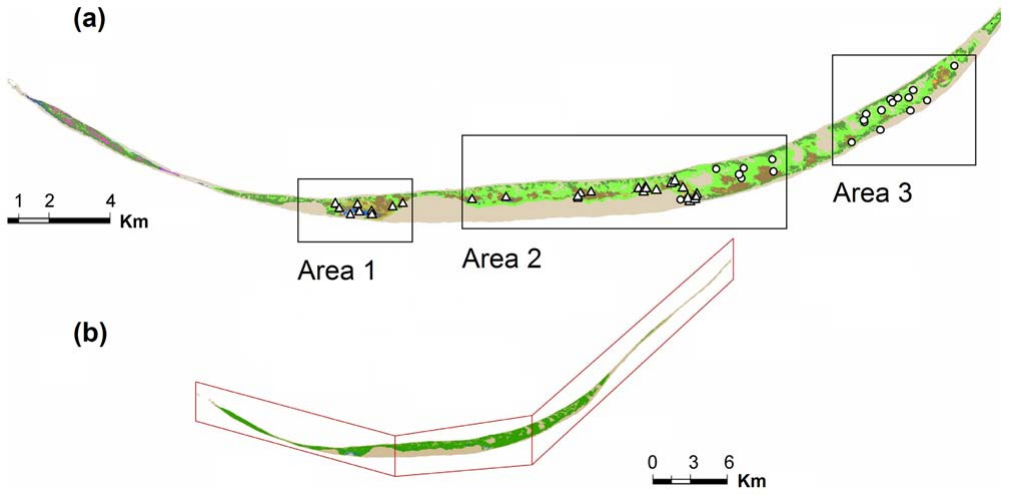
Study area and longitudinal gradient in sources of fresh water for feral horses on Sable Island, Canada, 2008–2010. (a) Sources of water (*n* = 122) grouped according to hierarchical cluster analysis, with area 1 containing only permanent ponds (triangles); area 2 containing permanent ponds and horse-excavated wells (circles); and area 3 containing only excavated wells. (b) Boundaries for areas 1, 2, and 3 that include clusters of water sources and the exclusive movements of females using those water sources.

In 2008, we initiated an individual-based research project on the ecology and evolution of the feral horses living on Sable Island, Nova Scotia, Canada (43°55′N, 60°00′W). The island is a crescent-shaped sand bar located 275 km southeast of Halifax (total area is approximately 3,000 ha [16]; Fig. 1). Sable Island's climate is temperate oceanic. Normal winter temperatures range from −10°C to 5°C, with maximum summer temperatures of 25°C [17]. Yearly precipitation averages 124 cm, with snow accounting for only 9% of the total [17].

Topography is heterogeneous and includes wide beaches and grassy plains, hummocky heaths, and vegetated and bare sand dunes up to 30 m in height. Sable Island's plant associations are limited mostly to grasses that dominate approximately 40% of the island area. A full description of the flora of Sable Island is presented by Catling et al. [18] and Stalter and Lamont [19]. Disturbance gradients associated with distance from shore structure the vegetation communities of the island [20], which are also affected by horse grazing [21]. Species with traits better suited to withstand sand burial and salt spray occur in near-shore areas (e.g., sandwort, *Honckenya peploides*, and marram, *Ammophila breviligulata*), while expanses of marram and associations of marram-forb (including beach pea, *Lathyrus japonicus* var. *maritimus*) and marram-fescue (with *Festuca rubra*) dominate the inland vegetated area. The most protected areas support diverse shrub and heath communities (including species like *Empetrum nigrum* and *Juniperus communis* var. *megistocarpa*). The island has no trees.

Permanent water ponds used by horses are confined to western and central areas of the island and cover only 21.8 ha in total [16]. Ephemeral melt- and rain-water ponds occur in the east half of the island but these generally disappear in mid-summer. Horses in the eastern part of the island must dig wells in the sand below the water table to access fresh water [16].

### Horse population

Aside from a small human presence (researchers, tourists, and staff at the island's meteorological station), the horses are the only terrestrial mammal on the island. Originally introduced in the mid-1700s, Sable Island's feral horses have always been free-ranging with minimal interference by humans [22], [23], [24]. The horses exhibit a mating system characterized by female-defense polygyny with persistent, non-territorial breeding groups (bands), which include a harem of adult females (mares), their pre-dispersing offspring, and 1–3 adult males (stallions). Other social groupings include mixed adolescent groups, all-male associations (‘bachelor’ groups), and solitary individuals. The Sable Island horse presents a markedly distinct genetic structure from other horse breeds and is likely of mid-18th century Acadian (eastern French Canadian) origin. The population is most closely related to the Nordic pony breeds [24], [25], [26].

In 2008, 2009, and 2010 we carried out a systematic ground (walking) survey of the entire horse population during summer (*n* = 484 individuals in 2010), concentrating our efforts in the breeding season (June–September). During these times, we conducted island-wide surveys once per 8–10 days by dividing the island into eight sections, and sampling first the odd-numbered then even-numbered sections in daily succession. Horses were easily observed from a distance, and though feral, most could be approached to within a few meters. Each sampling observation consisted of a horse location recorded on foot using a hand-held Global Positioning System (GPS) or combined digital camera-GPS unit, and information on the identity, sex, field age, band association, and reproductive status of the horse. We assessed mortality whenever horses disappeared from the island from one year to the next. We also scored body condition for females when encountered using the visual 5-point scale (11 levels with half points) of Carroll and Huntington [27]. We photographed individuals for identification from unique markings, facial portraits, and coloration patterns. Few horses were missed during a summer of sampling. We confirmed this in 2010 by comparing our ground survey results to that of an aerial photographic census, which revealed that we had counted >99% of the animals present. We estimated horse ages in the field based on size and appearance as foals (age 0), yearlings (age 1), subadults (ages 2 or 3), or adults (ages 4+). We verified that our field ages were generally without error by comparing ages collected in the field for 2008 yearlings (foals in 2007) as they entered the adult age category in 2011 (our surveys continue into the present day). In this study we focus on analysis of the female component of the population only, as we knew 98.7% (236 of 239) of encountered females by repeated observation.

### Resource availability

As part of our ground surveys, we recorded all locations with a GPS where we observed horses drinking fresh water. These included ponds but also points where horses accessed water by excavating (digging) wells to the water table. To document spatial trends in availability of different types of plant forage, we sampled vegetation along the length of Sable Island concurrent with our horse surveys between 16 July and 2 September 2010 using a sampling design corresponding to the island sections used in horse surveys. For each section, we randomly selected three north-south transects (24 in total) which we used to position vegetation plots. The order of sampling individual transects was mixed across the island, similar to the sampling order for horse surveys. Transects were divided into zones based on distance from shore, including zones classed as near shore (0–100 m), far shore (100–250 m), and central (>250 m). We sampled one plot per zone and transect (*n* = 135 plots). Each plot consisted of a 2 m^2^ circle around a centre point, divided into four quarters that were sampled with randomly positioned 0.5 m^2^ quadrats that were sectioned as four equal quarters. Vascular plant species were given a score from 0–4 according the presence or absence of a species within the four quarters of the quadrat. Quadrat scores were summed for a plot, with zero the minimum score possible and 16 the maximum. Species present in the 2 m circle of a plot, but not in any of the four quadrats, received a score of zero. We also estimated the total percent cover of vegetated areas. Horses were only ever observed using non-vegetated (beach or 100% sand) areas when travelling or eating seaweed. Hence, we calculated the mean percent vegetation cover for each island section to compare species and total vegetation abundance across the island only for plots with vegetation cover >0%.

### Population stratification based on resource availability

How the population might stratify based on spatial heterogeneity in resources was not known. However, it made sense that horses would respond to spatial differences in water availability. Heterogeneity of water as a resource is known to influence spatial distribution and movement patterns for several large herbivores (e.g., Rubenstein [28] and Berger [29] for feral horses; Ritter and Bednekoff [30] for Springbok *Antidorcas marsupialis*; Chamaillé-Jammes et al. [31] for African elephant *Loxodonta africana*). Therefore, we used locations where horses obtained water as the basis for spatially stratifying the population into distinct subunits. We chose a polythetic agglomerative hierarchical cluster analysis (HCA) to group Cartesian locations (*n* = 122) of water sources because the method accurately identifies spatially distinct groups nested within a total population [1], [32]. We used a Euclidean distance dissimilarity matrix calculated from unstandardized location data because we were interested in grouping water sources based on space, in terms of scale and dimensions, and standardization would remove this information [33]. Starting with all water locations as separate points, HCA formed groups by fusion based on similarity of locations [32]. We grouped water sources using average-linkage [1], [34], which considered mean distance between resources in one group with all resources in another. We produced a dendrogram to display results of our HCA, where distance between water-source groupings was represented by the height of the lines connecting groups [32]. We determined the most appropriate number of groups (3-group solution) from a *K*-means analysis [32], [35].

We applied an indicator-species analysis [36] to our plant-species data (*n* = 33 species) to determine if there were any plants that showed distinct patterns of abundance across the *a priori* defined groups of water availability determined by HCA. Significant indicator species are species highly characteristic of that *a priori*, whereas a perfect indicator of a group is one that is always present and exclusive to that group [36]. Using the “labdsv” package [37] in R [38] we calculated indicator values (IVs) for species from relative abundance and frequency of data for each species in each group. An indicator species has a high IV and low probability (*P*<0.05) of obtaining an IV of equal of higher value by chance [32]. We tested IVs for significance using 1000 permutations of a Monte Carlo simulation.

After stratifying the island based on water-group clusters and testing for associated IVs of vegetation, we super-imposed locations of horses (on an *x*-*y* scale [UTM zone 20]) onto clusters to determine if horses could be grouped in the same manner as did water and indicator plant species. Using a Geographical Information System (GIS), we confirmed whether identified areas (regions) matched movements of females within each year, and fixed boundaries (by longitudinal lines; Fig. 1b) such that females that used any particular area in a year had movements that were exclusively contained within the area. Our process thus made no assumptions of how the horse population might cluster based on horse movements, but rather stratified the population based on the location and types of resources that horse movements were expected to cluster upon (water and vegetation).

### Area-specific demography

For each year, females found in a given area were assigned as residents. We estimated annual population size for each area from count data. We calculated density as the total number of females per km^2^ of vegetated area observed during the sampling year. We computed the annual, realized rate of population increase (λ) for each area by dividing the population size (*N*) at year *t*+1 by that of the year previous 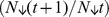 where values of λ>1 indicates population expansion, λ<1 indicates population decay, and λ = 1 indicates population stability [39]. We present annualized population growth as the geometric mean of both animal years of study.

We calculated apparent, age-specific survival and fecundity rates for each area by considering losses due to deaths/emigration and gains due to births/immigration. For each animal year (*t* to *t*+1), we noted a female as dead when not observed anywhere on the island at *t*+1, and an emigrant when alive at *t*+1 but located in a different area. Annual rates were calculated from total count data. We calculated apparent survival for each area as the proportion of residents from censuses at *t* who were observed alive in the same area at *t*+1. We present apparent survival for the study as the geometric mean among years.

For each area, we assigned foals to residents or immigrants and estimated annual fecundity as the number of surviving foals for a female of age-class *i* at *t*+1. We computed age-specific fecundity rates as the average of individual values, using a post-birth pulse calculation: a female's survival probability times the number of yearlings she was expected to contribute to the next year's census at *t* [39], [40]. We present apparent fecundity for the study as the geometric mean among years.

Using the R package “MASS” [41], we developed log-linear models to test whether apparent survival and fecundity differed among identified areas during our study [39], [42]. We also tested for differences in annual body condition of females of different areas using a Mann-Whitney *U* test [43] using the R base package.

## Results

### Population stratification

**Figure 2:**
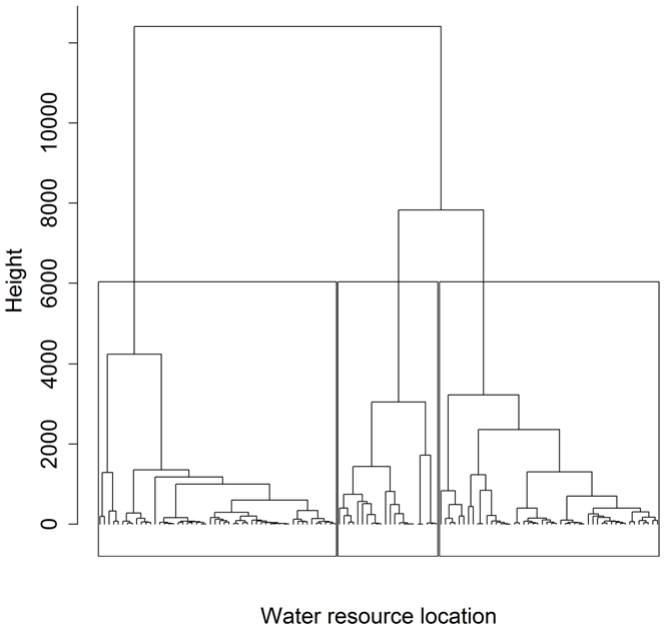
Three-group dendrogram showing average clustering of Cartesian locations of water sources (permanent water ponds and horse-excavated wells, *n* = 122) on Sable Island, Canada, 2008–2010. Boxes separate groups; each node represents a single location of a water source. The height of lines connecting water sources represents the distance between groupings. The left-most box contains sources located in area 2; the centre box contains sources located in area 1; the right-most box contains sources in area 3.

**Figure 3:**
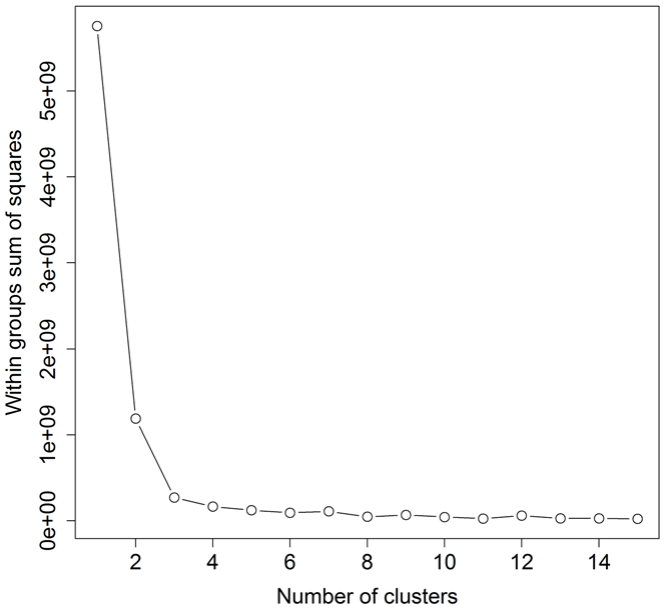
Graphical results of a *K*-means analysis indicating the appropriate number of clusters of water source-groupings, based on within-groups sum of squares as a function of the number of clusters from hierarchical cluster analysis of points of water use (*n* = 122), for the analysis of water availability to the Sable Island horses, 2008–2010. The first obvious bend indicates the appropriate number of groups (i.e., 3).

**Figure 4:**
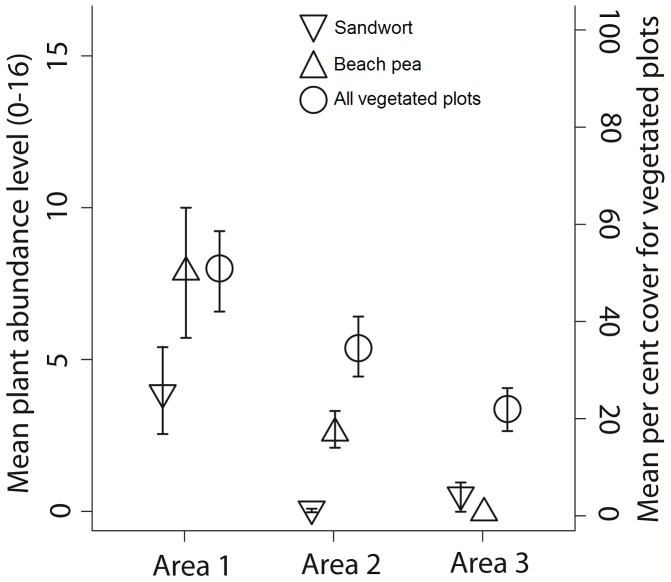
Comparison of the mean abundance of sandwort *Honckenya peploides* (right-side-up triangle) and beach pea *Lathyrus japonicas* (upside-down triangle) with percent cover for vegetated plots (open circle) in areas 1, 2, and 3 (west to east) on Sable Island, Canada, 2010. **Error bars are ±1 SE.**

Our HCA and a *K*-means analysis suggested three distinct water-source groupings (Figs. 1, 2 and 3; Table 1). Female proximity to water groups defined borders for area 1 (west), area 2 (central), and area 3 (east). We identified two indicator species for area 1: sandwort (IV = 0.382, *P* = 0.003, 89% cover) and beach pea (IV = 0.429, *P* = 0.003, 75% cover). No indicator species were identified for areas 2 or 3. Sandwort and beach pea were found at higher frequency and abundance in area 1 compared to areas 2 and 3 (Fig. 4; Table 1). Sandwort was nearly absent from areas 2 and 3; we found no beach pea in our plots in area 3. Other dominant plant species such as marram and *Poa* had a relatively even distribution throughout the island. Shrub species like *Empetrum nigrum*, junipers, and *Calluna vulgaris* were only observed in plots of area 2, which is the widest region of the island and most protected from coastal stress. Use of defined areas 1, 2, and 3 by female horses was confirmed as near exclusive during a year. During each summer, all but four females remained residents of the area in which they were originally observed.

**Table 1:** Habitat features, water, percent cover, and abundance of indicator species for three island areas, from west to east, on Sable Island, Canada, (August 2010).

	Total surface area (km^2^)	Proportion of total island area	Proportion vegetated area	Water type and summer availability	Proportion surface water	Mean % cover (± SE) of vegetated plots	Mean abundance^a^ of sandwort per vegetated plot (± SE)	Mean abundance^a^ of beach pea per vegetated plot (± SE)
Area 1	7.98	0.264	0.468 (3.691 km^2^)	Permanent ponds throughout	0.016 (0.123 km^2^)	50.00 (±8.55)	3.86 (±1.56)	7.93 (±2.05)
Area 2	13.26	0.443	0.552 (7.317 km^2^)	Some permanent ponds throughout, some digging holes	0.007 (0.096 km^2^)	33.62 (±4.81)	0.03 (±0.03)	2.63 (±0.78)
Area 3	8.79	0.293	0.440 (3.867 km^2^)	No permanent ponds, digging holes only	-	21.10 (±4.73)	0.44 (±0.44)	0 (±0)

a Abundance measured on a 0–16 presence/absence scale per plot.

### Area-specific demography

**Figure 5:**
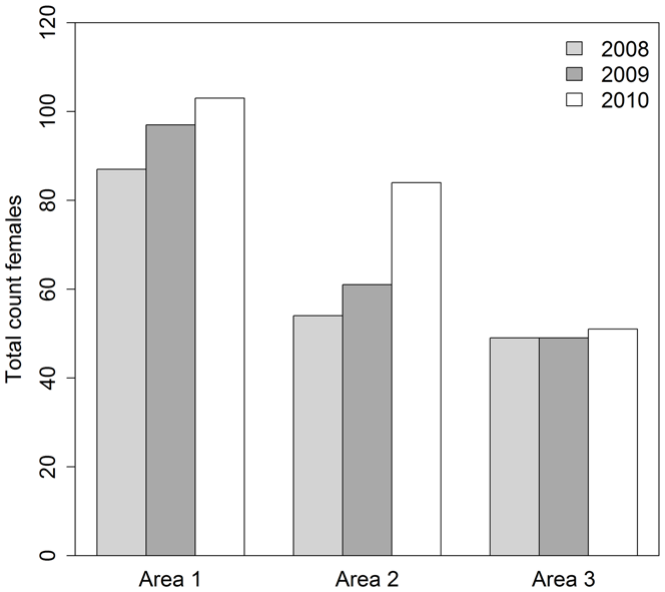
Comparison of the annual count in summer (August) of females of all age classes for areas 1, 2, and 3 (west to east) from 2008 to 2010, Sable Island, Canada.

We counted 265 females over our two year study. Age structure, expressed as average proportion in the local population, was relatively consistent between areas (Table 2). The number of females in each area increased during our study (Fig. 5). Average growth was most rapid in Area 2 (Table 3). Area 2 also had the lowest local density of females and produced the lowest number of emigrants; however, area 2 did accept the highest number of immigrants compared to other areas. Twenty-two over-winter immigration events were noted between 2008 and 2010.

**Table 2:** Demographic characteristics (annualized) of female horse for areas 1, 2, and 3 (west to east) for Sable Island, Canada (June–September 2008–2010).

	Age structure			Apparent survival^a^			Fecundity^b^		
Age class	Area 1	Area 2	Area 3	Area 1	Area 2	Area 3	Area 1	Area 2	Area 3
Foal	0.189	0.168	0.167	0.823	0.938	0.954	-	-	-
Yearling	0.127	0.121	0.128	0.857	0.667	0.894	-	-	-
2-year-old	0.125	0.106	0.154	0.885	1.000	0.732	-	-	-
3-year-old	0.113	0.101	0.101	0.805	0.849	0.645	0.140	7.457^−06^	0.232
Adult	0.447	0.503	0.449	0.897	0.894	0.862	0.311	0.240	0.133

a Annualized survival rates consider emigration from a subunit at *t*+1 as death, and are calculated for females only.

a,b Annualized survival and fecundity are calculated as geometric mean.

**Table 3:** Density and population growth characteristics of female horses in areas 1, 2, and 3 (west to east) on Sable Island, Canada (June–September 2008–2010).

	Average local density^a^ (no. females per vegetated km^2^)	Total emigrants	Total immigrants	Δ population size	λ_ann_ b
Area 1	25.06	10	5	16	1.088
Area 2	8.95	3	15	30	1.247
Area 3	12.80	9	2	2	1.020

a Arithmetic mean.

b Annualized population growth calculated as geometric mean.

Local density was highest in area 1, and most emigrants left from this area (Table 3, Fig. 5). By 2010, area 1 had increased in size by 16 females, but average growth was slower compared to area 2 (Table 3). Females of area 3 remained near stability (Table 3), only increasing by two females during our study (though number of males increased considerably [data not shown]). Average local density of females in area 3 was nearly half that of area 1 (Table 3), despite having near similar vegetated surface areas (Table 1). Even with these differences, the number of females leaving area 3 (9) was almost equal to those leaving Area 1 (10).

Log-linear models indicated there was no effect of age class and area on apparent survival and fecundity (Table 2) during our study (*G^2^* = 1.550, df = 2, *P* = 0.461 and *G^2^* = 5.614, df = 2, *P* = 0.060, respectively). Foals and yearlings tended to survive best in area 3; however, apparent survival for all other age classes was lowest in this area. Yearlings had the lowest chance of survival in area 2, but by age 2 residents enjoyed comparably high rates of survival. Apparent survival was most consistent in area 1. Average reproduction was highest for adults in area 1.

Over all three years, females living in area 1 had better body condition compared to females living in areas 2 (Mann-Whitney *U* test; *P* = 0.033) and 3 (Mann-Whitney *U* test; *P* = 0.001). There was no difference in condition between females of areas 2 and 3 (Mann-Whitney *U* test; *P* = 0.184).

## Discussion

Although Lucas et al. [13] quantified genetic substructure in the Sable Island horses across four arbitrary divisions of the island, we believe that availability of water and food resources leads to three defensible groupings divided in longitudinal fashion. That said, our structuring of the population does not differ markedly from that of Lucas et al. [13], aside from collapsing subdivisions II and III of Lucas et al. into our single area 2. Our area 1 is exactly the same as subdivision I of Lucas et al. [13]; area 2 is located in Lucas et al.'s subdivision II (which, as in our study, was noted to contain the last permanent water ponds when moving east along the island: Lucas et al. [13]: p. 314) and most of Unit III; and our area 3 accounts for the majority of Lucas et al.'s subdivision IV. Lucas et al. [13] found 10% greater heterozygosity in subdivision IV compared to subdivisions I, II, and III, suggesting outbreeding in eastern subdivision IV and inbreeding in western subdivisions (I, II, and III). This is consistent with a spatial distinction between area 3 and areas 1 and 2. Increased effective number of alleles and allelic richness between subdivisions I and III [13] are consistent with spatial dissimilarity between areas 1 and 2. That Lucas et al. found no significant difference in *F*_ST_ values between subdivisions III and IV corroborates our boundary between areas 2 and 3 falling within subdivision III.

We were concerned that our area boundaries might be viewed as arbitrary, given that habitat barriers do not obviously exist on Sable Island, and so could not explain any irregularities in horse movements or demographics. However, the population is clearly not homogenously distributed in space, given that local density is consistently highest in area 1 and lower in the other regions. Our hierarchical cluster analysis independently identified three spatial clusters in the gradient of water availability, and both the scale and positioning of these clusters corresponds well to spacing behaviour for three groupings of females. Although HCA is an established method for identifying socio-ecological groups [44], [45], [46], it has recently gained popularity for studies of spatial substructure, especially where scale for population stratification is unknown [1], [34], [47].

On Sable Island, each grouping of horses found using one of the three water-source clusters was exposed to different availabilities of beach pea and sandwort, which we identified as indicator species for area 1 and are preferred forage species for Sable Island horses [22]. There was no complete mixing of the horse population during summer months, and we tended to relocate females in previously occupied areas in consecutive seasons. This suggests that the underlying mechanism limiting female summer movements involves clustering of resources important for reproduction, which we identify here as a west-east gradient in access to fresh water and availability of beach pea and sandwort.

We found that inbreeding detected by Lucas et al. [13] in western areas of the island was associated with high surface water availability, abundance of beach pea and sandwort, and local density. As the incidence of inbreeding increases with close spatial associations between relatives [7], it is not surprising we find high inbreeding coefficients in area 1. Although survival and fecundity for adults was highest in area 1, local population growth was slower compared to area 2. Slow growth speaks to the high number of emigrations from area 1, and may indicate that horses in the west are nearing carrying capacity. At high density, monopolization of mating and food by some females may have a negative effect on the performance of other females. Thus, some females can be expected to emigrate east where local densities are lower, but surface water, beach pea, and sandwort are less abundant, and shrub-heath species are more abundant. Increasing local density in one area can cause animals to occupy other areas where resources are of poorer quality [48], [49]. If high local density and associated inbreeding are affecting female performance in area 1, then perhaps emigration is used to increase chances of survival and reproduction. Natal dispersal in horses (i.e., dispersal of offspring from their birth band) is a strategy for inbreeding avoidance [50], [51], and could explain why a large number of females emigrate from area 1. Although our data on productivity and dispersal are limited, we can conclude that spatial patterns in density, survival, reproduction, and migration do not suggest any problems concerning inbreeding depression in Sable Island horses.

Outbreeding in the eastern area 3 (see [13]) is associated with no surface water availability, low abundance of beach pea and sandwort, and moderate local horse density. We found that annualized growth of females, resulting from low survival and fecundity, was slowest in this area (however, we note here that when we add information on males to the analysis, total population growth rate was much higher at λ = 1.17, compared to total [male+female] population growth in area 1, λ = 1.09, and area 2, λ = 1.16). Population dynamics in eastern Sable Island, where outbreeding is indicated, may be driven more by short-term movements (emigration, immigration) rather than resident female production, as compared to western Sable Island.

We are limited in being able to interpret results or trends concerning area-specific variation in age structure, survival, and reproduction given the short duration of our study, and include this data for completeness. That said, our results are in agreement with research on other feral horse populations that show survival and reproductive success for females is linked to body condition during the summer months [52]. On Sable Island, survival and reproduction of females was highest in area 1 where resources were most abundant. Residents of this region were also of the best body condition. In addition, there was no difference between body condition of females in areas 2 and 3, where resources were less abundant compared to area 1.

Our results indicate that the influence of habitat on individual performance, which translates into differences in local demography between areas, is linked to the fine-scale genetic structure of the Sable Island horse population [13]. Related horses shared habitat, population dynamics, as well as genes. We conclude that where habitat gradients exist, we can look for and expect fine-scale heterogeneity in population dynamics and genetics.

## References

[1] Coulson T, Albon S, Guinness F, Pemberton J, Clutton-Brock T (1997) Population substructure, local density, and calf winter survival in red deer (*Cervus elaphus*). Ecology 78: 852–863.

[2] Coltman DW, Pilkington JG, Pemberton JM (2003) Fine-scale genetic structure in a free-living ungulate population. Mol Ecol 12: 733–742. 12675828 10.1046/j.1365-294x.2003.01762.x

[3] Nussey DH, Coltman DW, Coulson T, Kruuk LEB, Donald A, et al (2005) Rapidly declining fine-scale spatial genetic structure in female red deer. Mol Ecol 14: 3395–3405. 16156811 10.1111/j.1365-294X.2005.02692.x

[4] Brazeau DA, Sammarco PW, Atchison AD (2011) Micro-scale genetic heterogeneity and structure in coral recruitment: fine-scale patchiness. Aquat Biol 12: 55–67.

[5] Cullingham CI, Merrill EH, Pybus MJ, Bollinger TK, Wilson GA, et al (2011) Broad and fine-scale genetic analysis of white-tailed deer populations: estimating the relative risk of chronic wasting disease spread. Evol Appl 4: 116–131. 25567957 10.1111/j.1752-4571.2010.00142.x PMC3352516

[6] Stopher KV, Walling CA, Morris A, Guinness FE, Clutton-Brock TH, et al (2012) Shared spatial effects on quantitative genetic parameters: accounting for spatial autocorrelation and home range overlap reduces estimates of heritability in wild red deer. Evolution Article first published online 9 April 2012 DOI: 10.1111/j.1558-5646.2012.01620.x. 10.1111/j.1558-5646.2012.01620.x PMC3437482 22834741

[7] Chesser RK (1998) Relativity of behavioral interactions in socially structured populations. J Mammal 79: 713–724.

[8] Veran S, Beissinger SR (2009) Demographic origins of skewed operational and adult sex ratios: perturbation analyses of two-sex models. Ecol Lett 12: 129–143. 19143825 10.1111/j.1461-0248.2008.01268.x

[9] Fitze PS, Le Gailliard J-F (2011) Inconsistency between different measures of sexual selection. Am Nat 178: 256–268. 21750388 10.1086/660826

[10] Chesser RK (1991) Gene diversity and female philopatry. Genetics 127: 437–447. 2004714 10.1093/genetics/127.2.437 PMC1204371

[11] Thouless CR, Guinness FE (1986) Conflict between red deer hinds: the winner always wins. Anim Behav 34: 1166–1171.

[12] Fortin D, Morris DW, McLoughlin PD (2008) Adaptive habitat selection and the evolution of specialists in heterogeneous environments. Isr J Ecol Evol 54: 311–328.

[13] Lucas ZL, McLoughlin PD, Coltman DW, Barber C (2009) Multiscale analysis reveals restricted gene flow and a linear gradient in heterozygosity for an island population of feral horses. Can J Zool 87: 1–7.

[14] Wright S (1965) The interpretation of population structure by F-statistics with special regard to systems of mating. Evolution 19: 395–420.

[15] Weir BS, Cockerham CC (1984) Estimating F-statistics for the analysis of population structure. Evolution 38: 1358–1370. 28563791 10.1111/j.1558-5646.1984.tb05657.x

[16] Contasti AL (2011) Structure in vital rates, internal source-sink dynamics, and their influence on current population expansion for the feral horses (*Equus ferus caballus*) of Sable Island, Nova Scotia. M.Sc. Thesis, Department of Biology, University of Saskatchewan.

[17] Environment Canada (2011) National climate data and information archive. Available: http://www.climateweatheroffice.gc.ca Accessed 2012 Sept 28.

[18] Catling PM, Freedman B, Lucas Z (1984) The vegetation and phytogeography of Sable Island, Nova Scotia. PNSIS 34: 181–247.

[19] Stalter R, Lamont E (2006) The historical and extant flora of Sable Island, Nova Scotia, Canada. J Torrey Bot Soc 133: 362–374.

[20] Tissier EJ (2011) Vegetation associations along disturbance gradients on the sand dunes of Sable Island, Nova Scotia. M.Sc. Thesis, Department of Biology, University of Saskatchewan.

[21] Freedman B, Catling PM, Lucas ZL (2011) Effects of feral horses on vegetation of Sable Island, Nova Scotia. Can Field-Nat 125: 200–212.

[22] Welsh DA (1975) Population, behavioural, and grazing ecology of the horses of Sable Island, Nova Scotia. Dissertation, Dalhousie University.

[23] Christie BJ (1995) The Horses of Sable Island. Nova Scotia: Pottersfield Press.

[24] Plante Y, Vega-plas JL, Lucas Z, Colling D, De March B, et al (2007) Genetic diversity in a feral horse population from Sable Island, Canada. J Hered 98: 594–602. 17855732 10.1093/jhered/esm064

[25] Prystupa JM, Juras R, Cothran EG, Buchanan FC, Plante Y (2012a) Genetic diversity and admixture among Canadian, Mountain and Moorland, and Nordic pony populations. Animal 6: 19–30. 22436150 10.1017/S1751731111001212

[26] Prystupa JM, Hind P, Cothran EG, Plante Y (2012b) Maternal lineages in native Canadian equine populations and their relationship to the nordic and mountain and moorland pony breeds. J Hered 103: 380–390. 22504109 10.1093/jhered/ess003

[27] Carroll CL, Huntington PJ (1988) Body condition scoring and weight estimation of horses. Equine Vet J 20: 41–45. 3366105 10.1111/j.2042-3306.1988.tb01451.x

[28] Rubenstein DI (1981) Behavioural ecology of island feral horses. Equine Vet J 13: 27–34.

[29] Berger J (1986) Wild Horses of the Great Basin. Chicago: University of Chicago Press.

[30] Ritter RC, Bednekoff PA (1995) Dry season water, female movements and male territoriality in springbok: preliminary evidence of waterhole-directed sexual selection. Afr J Ecol 33: 395–404.

[31] Chamaillé-Jammes S, Valeix M, Fritz H (2007) Managing heterogeneity in elephant distribution: interactions between elephant population density and surface-water availability. J Appl Ecol 44: 625–633.

[32] McCune B, Grace JB (2002) Analysis of Ecological Communities. MjM Software Design, Gleneden Beach, Oregon.

[33] Kenkel NC (2006) On selecting an appropriate multivatriate analysis. Can J Plant Sci 86: 663–667.

[34] Mauritzen M, Derocher AE, Wiig Ø, Belikov SE, Boltunov AN, et al (2002) Using satellite telemetry to define spatial population structure in polar bears in the Norwegian and western Russian Arctic. J Appl Ecol 39: 79–90.

[35] van Sickle J (1997) Using mean similarity dendrograms to evaluate classifications. J Agric Biol Environ Stat 2: 370–388.

[36] Dufrêne M, Legendre P (1997) Species assemblages and indicator species: The need for a flexible asymmetrical approach. Ecol Monogr 67: 345–366.

[37] Roberts DW (2010) labdsv: Ordination and multivariate analysis for ecology. R package version 1.4-1 Available: http://CRAN.R-project.org/package=labdsv.

[38] R Development Core Team (2011) R: A language and environment for statistical computing. R Foundation for Statistical Computing, Vienna, Austria. ISBN 3-900051-07-0. Available: http://www.R-project.org/.

[39] Caswell H (2001) Matrix population models construction, analysis, and interpretation, Sinauer Associates, Inc. Sunderland, Massachusetts, USA.

[40] Morris WF, Doak DF (2002) Quantitative Conservation Biology theory and practice of population viability analysis. Sunderland: Sinauer Associates, Inc.

[41] Venables WN, Ripley BD (2002) Modern Applied Statistics with S. Fourth Edition. New York: Springer.

[42] Virgl JA, Messier F (2000) Assessment of source-sink theory for predicting demographic rates among habitats that exhibit temporal changes in quality. Can J Zool 78: 1483–1493.

[43] Siegel S, Castellan NJ Jr. (1988) Nonparametric statistics for the behavioural sciences. New York: McGraw Hill, Inc.

[44] Gadagkar R, Joshi NV (1983) Quantitative ethology of social wasps: time-activity budgets and caste differentiation in *Ropalidia marginata* (Lep.) (Hymenoptera: vespidae). Anim Behav 31: 26–31.

[45] Wittemyer G, Douglas-Hamilton I, Getz WM (2005) The socioecology of elephants: analysis of the processes creating multitiered social structures. Anim Behav 69: 1357–1371.

[46] Weise MJ, Harvey JT, Costa DP (2010) The role of body size in individual-based foraging strategies of a top marine predator. Ecology 9: 1004–1015. 10.1890/08-1554.1 20462115

[47] Edwards MA, Nagy JA, Derocher AE (2008) Using subpopulation structure for barren-ground grizzly bear management. Ursus 19: 91–104.

[48] Fretwell DS, Lucas HL (1969) On territorial behavior and other factors influencing habitat distribution in birds. Acta Biotheor 19: 16–32.

[49] Pulliam HR (1988) Sources, sinks, and population regulation. Am Nat 132: 652–661.

[50] Clutton-Brock TH (1989) Female transfer and inbreeding avoidance in social mammals. Nature 337: 70–71. 2909891 10.1038/337070a0

[51] Monard A-M, Duncan P, Boy V (1996) The proximate mechanisms of natal dispersal in female horses. Behaviour 133: 1095–1124.

[52] Cameron EZ, Linklater WL (2007) Extreme sex ratio variation in relation to change in condition around conception. Biol Lett 3: 395–397. 17439844 10.1098/rsbl.2007.0089 PMC2390657

